# Impact of hepatic steatosis on treatment response in nuclesos(t)ide analogue-treated HBeAg-positive chronic hepatitis B: a retrospective study

**DOI:** 10.1186/s12876-020-01289-w

**Published:** 2020-05-12

**Authors:** Yi-Cheng Chen, Wen-Juei Jeng, Chao-Wei Hsu, Chun-Yen Lin

**Affiliations:** 1 Department of Gastroenterology and Hepatology, Chang Gung Memorial Hospital, and University, Linkou, No 5, Fu Hsing Street, Guishan Dist, Taoyuan City, 33302 Taiwan, Republic of China; 2 College of Medicine, Chang Gung University, No.259, Wen Hua 1st Rd., Guishan Dist, Taoyuan City, 33302 Taiwan, Republic of China

**Keywords:** Chronic hepatitis B, Hepatic steatosis, HBeAg seroclearance, Nucleos(t)ide analogue treatment

## Abstract

**Background:**

The impact of hepatic steatosis (HS) on treatment response following nucleos(t)ide analogue (NA) treatment for chronic hepatitis B (CHB) patients has not been clearly elucidated. We aimed to investigate the difference in HBeAg seroclearance between NA-treated HBeAg-positive CHB patients with and without HS.

**Methods:**

We retrospectively recruited HBeAg-positive CHB patients receiving liver biopsy and NA monotherapy. The baseline clinical characteristics and cumulative incidence of HBeAg seroclearance were compared between patients with and without HS and age/gender-matched subgroup analysis was performed.

**Results:**

A total of 196 patients were enrolled from 2003 April to 2016 October. The mean age was 39.6 ± 11.2 years, 142 (72.4%) were males and 94 (48%) had histological evidence of HS. Median treatment duration and follow-up period were 24.3 months and 54.9 months, respectively. HBeAg seroclearance was achieved in 56/102 (54.9%) and 54/94 (57.4%) patients with and without HS, respectively (*p* = 0.830). The 5-year cumulative incidence of HBeAg seroclearance in patients with and without HS was 62.8 and 67.7% in overall population (*p* = 0.398) and 62.4 and 66.9% in age/gender-matched subgroups (*p* = 0.395), respectively. The rate of HBeAg seroclearance was comparable between patients with or without HS in different NA monotherapy (all *p* > 0.05).

**Conclusions:**

HS had no significant impact on HBeAg seroclearance in HBeAg-positive CHB patients with NA monotherapy during long-term follow-up.

## Background

Chronic hepatitis B virus (HBV) infection is an important worldwide public health burden. There are approximately 250 million patients infected by HBV in the world [1]. The global prevalence of nonalcoholic fatty liver disease (NAFLD) is around 25% [2], affecting 17 to 46% of adult population in Western countries and 8 to 54% in Asia [3, 4]. The prevalence of hepatic steatosis in HBV-infected patients has been found to be 14–76% [5, 6]. There has been reported that HBV infection is associated with a lower risk of fatty liver development [7–9] and that hepatic steatosis (HS) is inversely associated with HBV DNA levels [6, 10, 11] under the hypothesis of viral replication attenuation [12, 13]. Two studies in Taiwan have pointed out that moderate and severe HS were associated with increased odds ratio of HBsAg seroclearance [14] and body mass index (BMI) ≥30 kg/m^2^ was a significant predictor of HBsAg seroclearance [15]. However, the impact of HS on antiviral treatment response in CHB has not been clearly elucidated.

HBeAg seroconversion is one of the therapeutic goals in HBeAg-positive CHB patients. Past studies have shown an equivocal association between HS and treatment response to pegylated interferon in CHB patients [16–18]. Some studies in Asian regions have reported different results in HBeAg seroconversion under HS (by histology or noninvasive methods) in nucleos(t)ide analogues (NAs) treated CHB patients [19–23]. With such conflicting observations in the effect of HS on HBeAg seroconversion under NA treatment, we conducted a retrospective study to explore the impact of biopsy-proven steatosis on treatment response in NA-treated HBeAg-positive CHB patients.

## Methods

### Patients

This retrospective study would recruit HBeAg-positive patients who were scheduled for NA treatment between 2003 April to 2016 October in Chang Gung Memorial Hospital, Linkou branch. All the enrolled patients had received liver biopsy before NA monotherapy and were treated for at least 12 months. Patients with hepatocellular carcinoma, co-infection with hepatitis C virus (HCV), hepatitis D virus (HDV) or human immunodeficiency virus (HIV), autoimmune, alcoholic and drug-induced liver diseases were excluded.

In Taiwan, antiviral therapy is reimbursed in HBeAg-positive CHB patients under the criteria of (1) positive HBeAg > 3 months and alanine aminotransferase (ALT) ≥5x upper limit of normal (ULN); or (2) positive HBeAg > 3 months, ALT 2-5x ULN, and HBV DNA ≥20,000 IU/mL or positive HBcAg in histology. The duration of reimbursement has been extended to the time of HBeAg seroclearance with one-year consolidation therapy since 2017 January. Antiviral therapy can be reimbursed indefinitely in cirrhotic patients with HBV DNA > 2000 IU/mL regardless of ALT levels.

This study was approved by Institutional Review Board (IRB) of Chang Gung Memorial Hospital (IRB No. 201701168B0).

### Laboratory measurements and definitions of treatment response

The baseline clinical characteristics collected from the electronic medical records included aspartate aminotransferase (AST), ALT, total bilirubin, platelet count, HBeAg, anti-HBe, anti-HCV, anti-HDV, HBV genotype, HBV DNA and quantitative HBsAg (qHBsAg). Stored serums were retrieved for assays of HBV genotype, HBV DNA or qHBsAg for any incomplete data. HBV genotype was determined by polymerase chain reaction-restriction fragment length polymorphism of the surface gene of HBV. Serum HBV DNA was assayed by COBAS® AmpliPrep/COBAS® TaqMan® HBV Test, version 2.0 (lower limit of detection: 20 IU/mL, Roche Diagnostics, Mannheim, Germany). Serum HBsAg levels were quantified using the Roche Elecsys HBsAg II quant assay (detection limit, 0.05–52,000 IU/mL; Roche Diagnostics, Mannheim, Germany) according to the manufacturer’s instructions. HBeAg, anti-HBe, anti-HCV and anti-HDV were tested with enzyme immunoassay kit (Abbott Diagnostics, North Chicago, IL).

HBeAg seroclearance was defined as HBeAg loss with or without the presence of anti-HBe in serial tests [21] during treatment or within 12 months after discontinuation of treatment without clinical flare-up (ALT ≥2xULN and HBV DNA > 2000 IU/mL). Virological response (VR) was defined as a serum HBV DNA level < 20 IU/mL or undetectable after NA treatment. Patients with missing values or no stored serum available for assay of HBV DNA were regarded as non-VR. HBeAg reversion was defined as reappearance of HBeAg after HBeAg seroclearance. HBeAg-negative hepatitis was defined as persistent HBeAg negativity with HBV DNA > 2000 IU/mL and ALT increasing to >2x ULN. HBsAg seroclearance was defined as HBsAg negativity for at least 12 months with or without the presence of anti-HBs until the last visit [24].

### Histological assessment

HS was defined as the presence of steatosis in over 5% of hepatocytes according to the Brunt criteria [25]. Histological steatosis was categorized into score 0 to 3 [< 5%, 5–33% (mild), > 33–66% (moderate) and > 66% (severe)] [26]. The fibrosis score was graded by Metavir or Ishak scoring system [27, 28]. A fibrosis scores of 4 by Metavir or 5 and 6 by Ishak scores were considered cirrhosis.

### Statistical analysis

Continuous variables are expressed as means and standard deviations (S.D.) or medians and interquartile ranges (IQR) as appropriate after testing for normal distribution using the Kolmogorov-Smirnov test and are compared by independent Student’s *t*-test or Mann-Whitney-U test between the groups with and without HS. One-way ANOVA or Kruskal-Wallis H test was performed to compare the difference of clinical characteristics among patients with different degrees of HS. Categorical variables were presented as the number of cases (proportions) and compared by Chi-squared or Fisher’s exact tests when appropriate. The Kaplan-Meier method with the log-rank test was used to compare the cumulative rate of HBeAg seroclearance between patients with and without HS. The patients with and without HS would be matched by age (±1 year) and gender in 1:1 ratio for further analysis. Cox proportional hazards regression analysis was performed to find the associated predictors for HBeAg seroclearance. Variables with *p* < 0.1 in univariate analysis were further analyzed in multivariate analysis. Patients were censored at the time of antiviral retreatment or until the last follow-up visit. Statistical analysis was performed by Statistics Package for Social Science (SPSS) software (version 22.0, SPSS Inc., Chicago, IL, USA). A two-tailed *p* < 0.05 was considered statistically significant.

## Results

### Baseline clinical characteristics

A total of 196 consecutive HBeAg-positive patients were enrolled in this study. The mean age was 39.6 ± 11.2 years, 142 (72.4%) were males, 159 (81.1%) were treatment-naïve, 94 (48%) had histological HS and 59 (30.1%) had cirrhosis. The NA treatment included lamivudine (LAM) in 75 (38.3%), adefovir dipivoxil (ADV) in 2 (1%), entecavir (ETV) in 61 (31.1%), telbivudine (LdT) in 46 (23.5%) and tenofovir disoproxil fumarate (TDF) in 12 (6.1%). Baseline clinical characteristics between patients with and without HS are shown in Table 1. Patients with HS were significantly older (42.7 vs. 36.2 years, *p* < 0.001), had higher BMI (25.2 vs. 22.7 kg/m^2^, *p* < 0.001), higher proportion of male gender (79.4 vs. 64.9%, *p* = 0.035), genotype C (57.4 vs. 35.5%, *p* = 0.004) and cirrhosis (39.2 vs. 20.2%, *p* = 0.006) than those without HS. The mean qHBsAg level was significantly lower in patients with HS than those without (3.7 vs. 4.0 log IU/mL, *p* = 0.009).

**Table 1:** Comparison of baseline clinical characteristics and treatment response between HBeAg-positive patients with and without hepatic steatosis (HS)

	Overall population				Age/gender-matched subgroups		
	Total	HS (−)	HS (+)	p	HS (−)	HS (+)	p
No	196	94	102		56	56	
Age at treatment, year	39.6 ± 11.2	36.2 ± 10.8	42.7 ± 10.8	< 0.001	38.5 (31–44)	39 (31–44)	0.930
Male	142 (72.4)	61 (64.9)	81 (79.4)	0.035	43 (76.8)	43 (76.8)	1.000
BMI, kg/m^2^	24.0 ± 3.2	22.7 ± 2.8	25.2 ± 3.1	< 0.001	23.0 ± 3.0	25.0 ± 3.0	0.003
Genotype				0.004			0.087
B	100 (53.5)	60 (64.5)	40 (42.6)		35 (62.5)	23 (44.2)	
C	87 (46.5)	33 (35.5)	54 (57.4)		21 (37.5)	29 (55.8)	
Treatment-naïve	159 (81.1)	77 (81.9)	82 (80.4)	0.929	43 (76.8)	45 (80.4)	0.818
Cirrhosis	59 (30.1)	19 (20.2)	40 (39.2)	0.006	13 (23.2)	17 (30.4)	0.522
AST, U/L	62 (42–89)	65 (42–108)	62 (42–84)	0.254	72 (45–137)	61 (38–84)	0.060
ALT, U/L	106 (68–167)	121 (75–211)	102 (62–153)	0.098	124 (79–216)	99 (68–154)	0.111
Total bilirubin, mg/dL	0.8 (0.7–1.0)	0.8 (0.7–1.1)	0.8 (0.6–1.0)	0.114	1.0 (0.7–1.2)	0.8 (0.6–1.0)	0.018
Platelet, 10^9^/L	190 (155–224)	198 (160–231)	183 (154–218)	0.141	190.8 ± 46.7	194.6 ± 45.7	0.676
qHBsAg, log IU/mL	3.8 ± 0.7	4.0 ± 0.6	3.7 ± 0.8	0.009	4.0 ± 0.6	3.8 ± 0.8	0.182
HBV DNA, log IU/mL	7.7 (6.8–8.3)	7.9 (7.1–8.3)	7.5 (6.5–8.1)	0.165	7.6 ± 1.0	7.4 ± 1.3	0.259
Antiviral treatment				0.676			0.961
LAM	75 (38.3)	40 (42.6)	35 (34.3)		24 (42.9)	22 (39.3)	
ADV	2 (1.0)	1 (1.1)	1 (1.0)		1 (1.8)	1 (1.8)	
LdT	46 (23.5)	23 (24.5)	23 (22.5)		12 (21.4)	15 (26.8)	
ETV	61 (31.1)	25 (26.6)	36 (35.3)		16 (28.6)	16 (28.6)	
TDF	12 (6.1)	5 (5.3)	7 (6.9)		3 (5.4)	2 (3.6)	
Treatment duration, m	24.3 (12.2–36.8)	24.3 (12.2–36.5)	23.7 (12.1–36.6)	0.306	23.8 (12.2–36.5)	24.3 (12.1–36.6)	0.835
Follow-up, m	54.9 (31.3–99.3)	59.6 (33.7–102.9)	45.2 (30.7–94.2)	0.145	55.1 (26.1–103.2)	50.9 (31.9–92.7)	0.979
**Treatment response**							
HBeAg seroclearance	110 (56.1)	54 (57.4)	56 (54.9)	0.830	30 (53.6)	28 (50)	0.850
Virological response	115 (58.7)	50 (53.2)	65 (63.7)	0.177	28 (50)	32 (57.1)	0.570
Age at e seroclearance	42.1 ± 11.7	38.5 ± 11.4	45.6 ± 11.0	0.001	41.5 ± 11.1	41.4 ± 10.2	0.959
Time to seroclearance, m	17.5 (9.3–34.8)	17.8 (8.9–28.6)	16.9 (9.4–39.9)	0.401	23.9 (15.4–38.8)	29.4 (16.0–45.5)	0.436
HBeAg reversion^a^	13 (11.8)	4 (7.4)	9 (16.1)	0.266	2 (6.7)	3 (10.7)	0.665
HBeAg(−) hepatitis^a^	35 (31.8)	15 (27.8)	20 (35.7)	0.491	9 (30)	10 (35.7)	0.854
HBsAg seroclearance	8 (4.1)	4 (4.3)	4 (3.9)	1.000	2 (3.6)	4 (7.1)	0.679

Data were presented as mean ± S.D. or median (interquartile range) and number (%)

Hepatic steatosis, histologic steatosis > 5%; *BMI* Body mass index; *AST* Aspartate aminotransferase; *ALT* Alanine aminotransferase; *qHBsAg* Quantitative HBsAg; *LAM*, lamivudine; *ADV* Adefovir dipivoxil; *LdT* Telbivudine; *ETV* Entecavir; *TDF* Tenofovir disoproxil fumarate

^a^Data analyzed based on HBeAg seroclearance

### Treatment response and associated factors for HBeAg seroclearance

After a median treatment duration of 24.3 months, HBeAg seroclearance was achieved in 110 (56.1%) patients (54 in non-steatosis and 56 in steatosis, *p* = 0.830) during a median follow-up period of 54.9 months. There were 40 (36.4%) patients with HBeAg seroclearance at the time of 12 months. Among those staying in this study without retreatment during follow-up, additional HBeAg seroclearance occurred in 29 (18.6%) at 24 months, 18 (17.8%) at 36 months, 13 (21%) at 48 months and 4 (11.4%) at 60 months (Supplementary Table 1). A total of 104 (53.1%) patients achieved HBeAg seroclearance at 5-year follow-up (anti-HBe occurred in 83 patients). The remaining 6 patients developed HBeAg seroclearance at 69.7, 78, 85.3, 88, 95.4, and 105.3 months of follow-up.

**Fig. 1:**
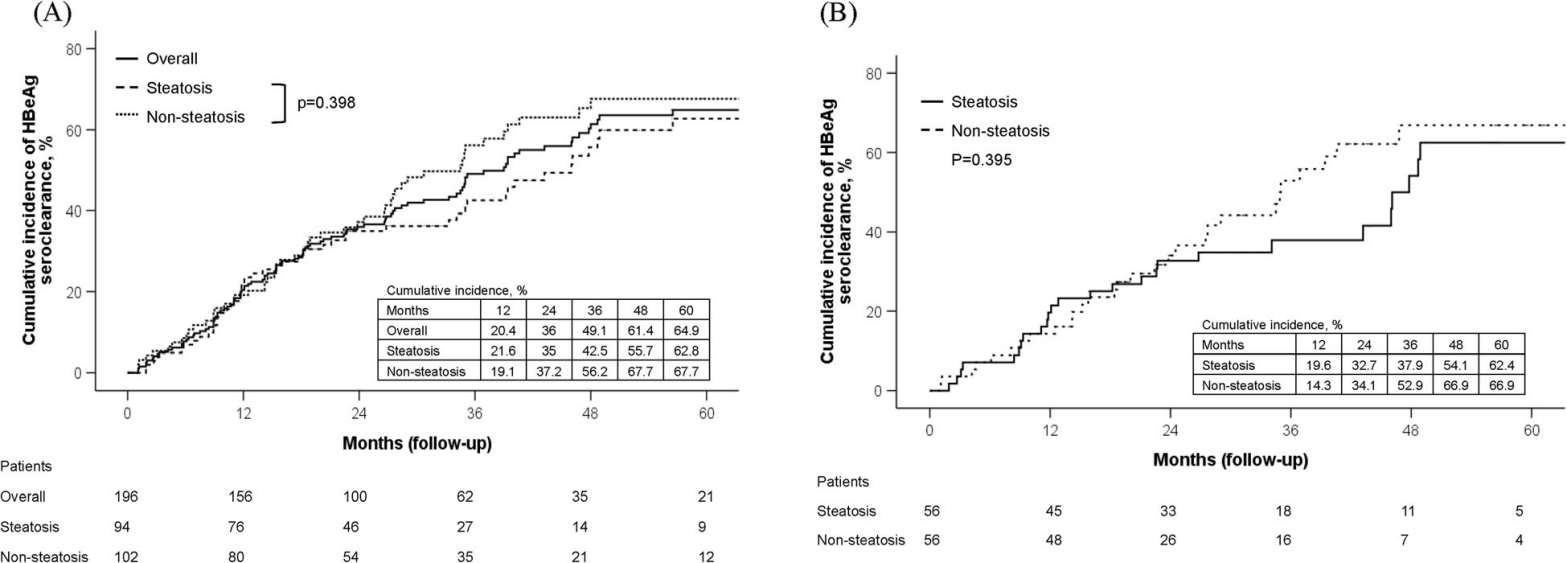
The cumulative incidence of HBeAg seroclearance in HBeAg-positive patients treated with nucleos(t)ide analogues by Kaplan-Meier analysis and log-rank test. (A) Comparison between patients with and without hepatic steatosis in overall population (*N* = 196). The 5-year cumulative incidence of HBeAg seroconversion was 62.8 and 67.7%, respectively (*p* = 0.398). (B) Comparison between patients with and without hepatic steatosis in age- and gender-matched subgroups (*N* = 112). The 5-year cumulative incidence of HBeAg seroclearance was 62.4 and 66.9%, respectively (*p* = 0.395)

The mean age at HBeAg seroclearance was significantly older in patients with HS than those without HS (45.6 vs. 38.5 years, *p* = 0.001), while the median time to HBeAg seroclearance was comparable between these two groups (16.9 vs. 17.8 months, *p* = 0.401) (Table 1). VR was achieved in 50 (53.2%) and 65 (63.7%) patients with and without HS, respectively (*p* = 0.177). There were 10 patients (2 HS and 8 non-HS) regarded as non-VR due to missing values for HBV DNA. After excluding these patients, VR was still not different between two groups (*p* = 0.419). The overall 5-year cumulative incidence of HBeAg seroclearance was 64.9%. The cumulative incidences of HBeAg seroclearance at 1 year, 3 years and 5 years were 21.6, 42.5 and 62.8% in patients with HS and 19.1, 56.2 and 67.7% in those without HS, respectively (*p* = 0.398 at 5 years, Fig. 1a). There was no significant difference in the rates of HBeAg reversion (*p* = 0.266), HBeAg-negative hepatitis (*p* = 0.491) and HBsAg seroclearance (*p* = 1.000) after HBeAg seroclearance between patients with and without HS (Table 1). There were 24 patients (20 cirrhosis) with continuous NA treatment until the last follow-up and 106 (54.1%) patients were retreated during follow-up period (12 HBeAg revision, 31 HBeAg-negative hepatitis and 63 persistent HBeAg-positivity with relapse).

HBeAg seroclearance occurred in 29 (38.7%), 44 (72.1%), 29 (63%), 0 and 8 (66.7%) in patients treated with LAM, ETV, LdT, ADV and TDF, respectively. There was no difference in HBeAg seroclearance between HS and non-HS patients in LAM (*p* = 0.334), ETV (*p* = 1.000), LdT (p = 1.000) and TDF (*p* = 0.576) treatment (Supplementary Table 2). The rate of VR was not statistically different between HS and non-HS patients in LAM (*p* = 0.381), ETV (*p* = 0.392), LdT (*p* = 1.000) and TDF (*p* = 1.000) treatment.

The Cox proportional hazard regression analysis was performed to determine the factors associated with HBeAg seroclearance by the baseline variables of age, gender, HBV genotype, treatment naïve, cirrhosis, HS, AST, ALT, total bilirubin, platelet count, antiviral treatment, qHBsAg and HBV DNA levels. The patients with ADV and TDF treatment were not included in the analysis because of small number of patients. In univariate analysis, gender (male vs female, hazard ratio [HR] 0.717, 95% confidence interval [CI] 0.482–1.064, *p* = 0.099), antiviral treatment (ETV vs LAM, HR 1.602, 95% CI 1.000–2.568, *p* = 0.050; LdT vs LAM, HR 1.841, 95% CI 1.098–3.087, *p* = 0.021), qHBsAg (HR 0.756, 95% CI 0.579–0.999, *p* = 0.040), and HBV DNA (HR 0.809, 95% CI 0.686–0.955, *p* = 0.012) were selected (*p* < 0.1) to be analyzed in multivariate analysis. Finally, only antiviral treatment (LdT vs LAM, HR 2.548, 95% CI 1.338–4.853, *p* = 0.004) and HBV DNA level (HR 0.729, 95% CI 0.546–0.972, *p* = 0.031) were the independent factors for HBeAg seroclearance (Table 2). HS was not an associated factor in both univariate and multivariate analyses.

**Table 2:** The associated factors for HBeAg seroclearance in the univariate and multivariate analyses

	Univariate		Multivariate	
Factors	HR (95% CI)	p	HR (95% CI)	p
Gender (M vs F)	0.717 (0.482–1.064)	0.099	0.667 (0.404–1.099)	0.112
Antiviral treatment				
LAM	reference		reference	
ETV	1.602 (1.000–2.568)	0.050	1.182 (0.660–2.118)	0.573
LdT	1.841 (1.098–3.087)	0.021	2.548 (1.338–4.853)	0.004
qHBsAg, log IU/mL	0.756 (0.579–0.999)	0.040	0.755 (0.515–1.107)	0.150
HBV DNA, log IU/mL	0.809 (0.686–0.955)	0.012	0.729 (0.546–0.972)	0.031
HS	0.851 (0.584–1.238)	0.399	0.680 (0.410–1.130)	0.137

Variables with *p* < 0.1 in univariate analysis were analyzed in multivariate analysis

*HR* Hazard ratio; *CI* Confidence interval; *qHBsAg* Quantitative HBsAg; *LAM* lamivudine; *LdT* Telbivudine; *ETV* Entecavir; *HS* Hepatic steatosis

### Clinical characteristics and treatment response in different degrees of hepatic steatosis

The comparison of baseline clinical characteristics and treatment response among 102 steatotic patients with different degrees of histological steatosis is shown in Supplementary Table 3. As expected, patients with mild steatosis had significantly lower BMI (24.3 kg/m^2^) than those with moderate steatosis (26.2 kg/m^2^, *p* = 0.043) and those with severe steatosis (27.9 kg/m^2^, *p* = 0.002) (*p* = 0.191 in overall comparison). The patients with severe steatosis had significantly lower mean HBV DNA level than those with mild steatosis (6.6 vs 7.6 log IU/mL, *p* = 0.036), while the difference was not significant (*p* = 0.531) as compared to the mean HBV DNA level (7.1 log IU/mL) of those with moderate steatosis. The other clinical characteristics were comparable among these three groups. HBeAg seroclearance was achieved in 37 (57.8%) with mild steatosis, 14 (56%) with moderate steatosis and 5 (38.5%) with severe steatosis (*p* = 0.438). The mean age at HBeAg seroclearance was 47.3, 42.6 and 40.8 years (*p* = 0.419) and the median time to HBeAg seroclearance was 17.7, 15.2 and 16 months (*p* = 0.898), respectively in the corresponding subgroups. The VR rate was similar among three groups (*p* = 0.875).

### HBeAg seroclearance in age- and gender-matched subgroups

Since the age at HBeAg seroclearance was significantly older in patients with HS in present study, an impression rose that HS would delay HBeAg seroclearance, like the observation in a previous study [21]. We therefore matched the patients with and without HS by age (±1 year) and gender in 1:1 ratio and there were 56 patients in each group for further analysis. The mean BMI was significantly higher in patients with HS than those without HS (25 vs 23 kg/m^2^, *p* = 0.003). The median total bilirubin level was significantly lower in patients with HS (0.8 mg/dL) as compared to that in patients without HS (1.0 mg/dL, *p* = 0.018). The other baseline clinical characteristics were comparable between two subgroups (Table 1). The HBeAg seroclearance rate (53.6 vs 50%, *p* = 0.850), mean age at HBeAg seroclearance (41.5 vs 41.4 years, *p* = 0.959) and median time to HBeAg seroclearance (23.9 vs 29.4 months, *p* = 0.436) were not statistically different between these two subgroups. HBeAg seroclearance occurred in 14 (30.4%), 26 (81.3%), 16 (59.3%), 0 and 2 (40%) in patients treated with LAM, ETV, LdT, ADV and TDF, respectively (*p* < 0.001). There was no significant difference in HBeAg seroclearance between patients with and without HS in LAM (*p* = 0.159), ETV (*p* = 0.654), LdT (*p* = 0.452) and TDF (*p* = 0.400) treatment (Supplementary Table 2). The 5-year cumulative incidences of HBeAg seroclearance were comparable between patients with and without HS (62.4 vs 66.9% at 5 years, *p* = 0.395) (Fig. 1b).

## Discussion

This is a real-world, retrospective study in Taiwan to discuss the impact of HS on HBeAg seroclearance in 196 HBeAg-positive patients with NA monotherapy based on the histological evidence, a gold standard for HS assessment [2, 3]. The prevalence of HS in this cohort was 52%, in accordance with those in previous reports ranging from 14 to 76% [5, 6]. The HBeAg seroclearance rate of 53% at 5 years was similar to that of 49% after 5-year TDF treatment [29]. According to the results, HS had no significant impact on treatment response in overall population and age/gender matched subgroups and among different NA monotherapy. This further confirmed the findings of previous studies using liver biopsy for HS evaluation [19, 21]. The studies discussing the influence of HS on HBeAg seroconversion/seroclearance under antiviral treatment were summarized in Table 3.

**Table 3:** Summary of studies discussing the influence of hepatic steatosis on HBeAg seroclearance/seroconversion under antiviral treatment

Source	Country	Hepatic steatosis (surrogate/method)	HBeAg(+) No	Treatment	HBeAg seroconversion/seroclearance
Charatcharoenwitthaya [19]	Thailand	Biopsy	38	IFN, LAM, ETV, TDF, LdT	non-steatosis 35% vs steatosis 27%, *p* = 0.599
Chung [20]	Korea	BMI ≥25 kg/m^2^	44	ETV	Normal BMI 36% vs BMI ≥25 kg/m^2^, *p* = 0.695
Hsiang [21]	Hong Kong	MetS	251 (124^a^)	ETV, TDF	Normal 39.7% vs pre-MetS 49.6% vs MetS 50%; HR 0.69 in steatosis ≥34%, *p* = 0.36
Jin [22]	China	Ultrasound	133	ETV	Steatosis 24.6% vs non-steatosis 28.4%, *p* = 0.13
Kim [23]	Korea	CAP	172	ETV, TDF	CAP < 238 dB/m 28.3% vs CAP ≥238 dB/m 13.8%, HR 0.991 in increasing CAP, *p* = 0.026
Present study	Taiwan	Biopsy	196	LAM, ADV, LdT, ETV, TDF	Non-hepatic steatosis 57.4% vs hepatic steatosis 54.9%, *p* = 0.830

*BMI* Body mass index; *CAP* Controlled attenuation parameter; *IFN* Interferon; *LAM* Lamivudine; *ADV* Adefovir dipivoxil; *LdT* Telbivudine; *ETV* Entecavir; *TDF* tenofovir disoproxil fumarate; *HR* Hazard ratio; *MetS* metabolic syndrome

^a^124 patients had received liver biopsy

As the NAFLD has become a common liver disease worldwide, the interaction between CHB and HS, as well as the associated metabolic syndrome and obesity, has been enthusiastically discussed recently. A pooled data in a systemic review [6] found a strong negative effect of HBV viral load on histology-proven HS [standardized mean difference − 74.12, 95% CI (− 82.91, − 65.31), *p* < 0.001]. An Indian study on 350 CHB patients showed that the median HBV DNA was significantly lower in those with biopsy-proven HS (6.9 × 10^5^ vs 7.5 × 10^6^ copies/mL, *p* = 0.025) [11]. A recent study in Hong Kong [10] using CAP for HS assessment also found that the median HBV DNA levels were significantly lower in steatotic treatment-naïve patients when compared to non-steatotic controls (2.8 vs 3.1 log IU/mL, *p* = 0.011). In our study, the patients with HS had lower median HBV DNA levels than non-HS patients but the difference was not statistically significant (7.5 vs 7.9 log IU/mL, *p* = 0.165). Inclusion of patients with all HBeAg positivity, most in the status of immune clearance phase and nearly one-third being cirrhosis may explain this discrepancy in the statistical analysis.

Of note is that the steatotic HBeAg-positive patients had significantly lower mean qHBsAg levels than non-steatotic patients (3.7 vs 4.0 log IU/mL, *p* = 0.009) in this study and this inverse relationship was rarely discussed in past literatures. As the HBsAg production reflects the replication of HBV DNA and active transcription of covalently closed circular DNA (cccDNA) [30], fat deposition in hepatocytes may inhibit viral replication and downregulate transcription of cccDNA and therefore decrease the HBV-related antigen expression and production [12], such as HBsAg protein. Although this discrepancy disappeared after age and gender adjustment, the patients with HS still had lower qHBsAg levels (3.8 vs 4.0 log IU/mL). Further and large-scaled studies are needed to explore this relationship. HBV genotype distribution was significantly different between patients with and without HS in overall population (*p* = 0.004). Even though the difference disappeared between age and gender-matched non-HS and HS subgroups (*p* = 0.087) (Table 1) which was coincident with previous reports [6, 11], the proportion of genotype C was still higher in patients with HS. This paradoxical distribution of genotype (C > B) as compared to general population (B > C) in Taiwan needs to be clarified by more studies.

In a Hong Kong study [10] using transient elastography for liver stiffness measurement (LSM) and controlled attenuation parameter (CAP), severe steatosis was associated with an increased percentage of severe fibrosis (23.2% vs 12.6%, *p* = 0.005) in CHB patients. Metabolic syndrome, which is strongly associated with NAFLD, has been reported to increase the risk of liver fibrosis progression and cirrhosis development by LSM (adjust OR 2.0 for fibrosis progression, *p* = 0.015; specificity 94% for cirrhosis) in CHB patients [31, 32]. These observations suggest that the co-existence of HS by indirect evidence can result in fibrosis progression in CHB patients. By contrast, the fibrosis staging was not different between CHB patients with or without histological HS in a meta-analysis including five studies (standardized mean difference 0.22, *p* = 0.495) [6]. The latter was further confirmed by the results in this study that patients with HS had significantly higher rate of liver cirrhosis (39.2 vs 20.2%, *p* = 0.006), but the difference was not significant between the age/gender-matched subgroups (30.4 vs 23.2%, *p* = 0.522). As histology is a gold standard for fibrosis staging in NAFLD, indirect evidence of HS like obesity, metabolic syndrome or CAP may be undesirable to specifically predict liver fibrosis in CHB patients and more validation is needed in the future. Regarding to the patients with different degrees of steatosis, there was no significant difference in the proportion of cirrhosis among mild, moderate and severe steatosis (36, 48 and 38.5%, respectively, *p* = 0.577) (Supplementary Table 3).

The impact of HS on treatment response to pegylated interferon in previous reports were controversial [16–18] and the association of HS with NA treatment response was also conflicting and has not been clearly elucidated so far. The study population in previous studies using liver biopsy for assessment of HS was number-limited and included patients with treatment by either pegylated interferon or NAs, both HBeAg-positive and HBeAg-negative or patients with antiviral treatment and treatment-free [19, 21]. In addition, some studies utilized indirect methods of BMI, ultrasound or CAP for clinical evaluation of HS [20, 22, 23]. By avoiding the heterogeneity in study population and study methods for HS assessment, this study was based on histological evidence of HS and recruited a larger number of HBeAg-positive patients (*n* = 196) and therefore could specifically explore the impact of HS on antiviral treatment response. The results came to comparable HBeAg seroclearance rate and no significant difference in the 5-year cumulative incidence of HBeAg seroclearance between patients with and without HS. VR was also comparable between patients with and without HS. Based on the histologic evidence of HS and a homogenous cohort, we believed that our findings could provide a reliable inference that there is no significant impact of HS on treatment response under NA monotherapy.

In line with previous randomized global studies [33, 34], HBeAg seroclearance rates were significantly higher in patients with ETV treatment (72.1%, *p* < 0.001) and LdT treatment (63%, *p* = 0.016) than those with LAM treatment (38.7%) in HBeAg-positive patients (Supplementary Table 2). In this study, both ETV and LdT were associated factors in HBeAg seroclearance relative to lamivudine in univariate analysis, while ETV, unlike LdT, lost this advantage in multivariate analysis (Table 2). This phenomenon could be explained by the heterogeneity of baseline clinical characteristics in patients with LAM, ETV and LdT treatment (supplementary Table 4). Older age and more patients with genotype C, cirrhosis and lower ALT were found in patients treated with ETV. In the results of this study, HS had no influence on the HBeAg seroclearance and VR among different NA treatment. In contrast to ETV, a recent study by Kim and his colleagues found the possibility of HBeAg loss was significantly lower in TDF-treated patients with hepatic steatosis [23]. Another study by Jin’s team showed a significantly increased rate of HBV DNA clearance in ETV-treated patients without HS but not the rate of HBeAg seroconversion [22]. A study including 145 biopsy-proven CHB patients, HS had no impact on VR to ETV and TDF treatment [35]. Decreased bioavailability of intrahepatic metabolites [36] of NAs due to hepatocellular fat droplet accumulation has been supposed be the reason for the different performance of NAs in treatment response. Diminished activity of hepatic cytochrome in steatotic hepatocytes, insulin resistance and obesity coexisted HS leading to dysfunction of cellular immune function may also affect the treatment outcomes [22]. More studies are needed to elucidate the influence of HS to NA treatment.

There are some limitations in our study. First, enrolled patients were limited to the conditions of HBeAg positivity, with liver biopsy and NA monotherapy and therefore the prevalence of hepatic steatosis or the clinical discrepancy between patients with or without HS or with different NA treatment could not represent the general population of CHB and might raise study bias. Nonetheless, we had the advantage of liver biopsy in all enrolled patients, which would be difficult to approach in future prospective studies as noninvasive methods have emerged recently. We also tried to minimize the clinical difference by matching age and gender between patients with and without HS. Second, the results about the impact of HS on HBeAg seroclearance with NA treatment should be validated in Western countries because all the previous reports and this study are restricted to Asia-Pacific regions with predominant genotype B and C. Third, there were insufficient data in metabolic profiles in our study for analysis. The effect of metabolic factors on treatment response is still a controversial issue. Although metabolic syndrome has been reported to delay HBeAg seroconversion [21], another study from Korea [37] showed that metabolic syndrome was not correlated with HBV DNA suppression and the cumulative rates of HBeAg negative conversion (*p* = 0.434) and seroconversion (*p* = 0.119) under NA treatment. Fourth, as the patient recruitment started in the early era of NA treatment and liver biopsy was no more a prerequisite for CHB treatment after approval of ETV and TDF in Taiwan, only 37% of patients were treated with ETV or TDF in this study cohort. Finally, only 10 patients had nonalcoholic steatohepatitis (NASH) assessment since NASH evaluation began in late 2014 in pathological reporting system. HBeAg positivity persisted in one with NASH and 7 of the remaining 9 patients without NASH achieved HBeAg seroclearance. It has been reported that NASH did not affect response to antiviral therapy of interferon or NAs [19].

## Conclusions

This study included a cohort of HBeAg-positive patients with histological evidence of HS under NA monotherapy and showed no significant difference in the cumulative incidence of HBeAg seroclearance between those with and without HS during long-term follow-up. This phenomenon was also observed in age/gender matched subgroups and patients with different NA monotherapy. Validation to the impact of HS on antiviral treatment response in HBV endemic areas with genotype non-B or non-C is warranted.

## Supplementary information

**Additional file 1: Supplementary Table 1.** Additional HBeAg seroconversion during long-term follow-up. **Supplementary Table 2.** HBeAg seroclearance in different nucleos(t)ide analogues with and without hepatic steatosis (HS). **Supplementary Table 3.** Comparison of baseline characteristics and treatment response among patients with different degrees of histological steatosis. **Supplementary Table 4.** Baseline clinical characteristics among patients with lamivudine (LAM), entecavir (ETV) and telbivudine (LdT) treatment*.

## Data Availability

The raw data collected and analyzed in the current study are not publicly available due to appropriate protection of patient personal information but are available from the corresponding author on reasonable request.

## Abbreviations

HS: Hepatic steatosis
NA: Nucleos(t)ide analogue
CHB: Chronic hepatitis B
HBV: Hepatitis B virus
NAFLD: Nonalcoholic fatty liver disease
BMI: Body mass index
HCV: Hepatitis C virus
HDV: Hepatitis D virus
HIV: Human immunodeficiency virus
ALT: Alanine aminotransferase
ULN: Upper limit of normal
AST: Aspartate aminotransferase
IQR: Interquartile ranges
LAM: Lamivudine
ADV: Adefovir dipivoxil
ETV: Entecavir
LdT: Telbivudine
TDF: Tenofovir disoproxil fumarate
HR: Hazard ratio
CI: Confidence interval
VR: Virological response
NASH: Nonalcoholic steatohepatitis
