# Supplementary material for: Impact of hepatic steatosis on treatment response in nuclesos(t)ide analogue-treated HBeAg-positive chronic hepatitis B: a retrospective study
Source: BMC Gastroenterol. 2020 May 12;20:146. doi: 10.1186/s12876-020-01289-w (PMC7216492; doi:10.1186/s12876-020-01289-w)
Supplement: Supplementary file 1 — Additional file 1: Supplementary Table 1. Additional HBeAg seroconversion during long-term follow-up. Supplementary Table 2. HBeAg seroclearance in different nucleos(t)ide analogues with and without hepatic steatosis (HS). Supplementary Table 3. Comparison of baseline characteristics and treatment response among patients with different degrees of histological steatosis. Supplementary Table 4. Baseline clinical characteristics among patients with lamivudine (LAM), entecavir (ETV) and telbivudine (LdT) treatment*. [file 12876_2020_1289_MOESM1_ESM.docx]

Supplementary Table 1. Additional HBeAg seroconversion during long-term follow-up

| Entry | Follow-up | 12M | 24M | 36M | 48M | 60M |
| --- | --- | --- | --- | --- | --- | --- |
| n=196 | HBeAg SC (+), n | 40 | 29 | 18 | 13 | 4 |
|  | HBeAg SC (−), n | 156 | 127 | 83 | 49 | 31 |
|  | Censored, n | 0 | 26 | 21 | 14 | 11 |
|  | (Additional) SC rate | 14% | 18.6% | 17.8% | 21% | 11.4% |

SC, seroclearance

* Patients were censored at retreatment or the last follow-up.

Supplementary Table 2. HBeAg seroclearance in different nucleos(t)ide analogues with and without hepatic steatosis (HS)

|  | HBeAg seroclearance | | | | | | | | | |
| --- | --- | --- | --- | --- | --- | --- | --- | --- | --- | --- |
| Overall, | LAM, n=75 | | ETV, n=61 | | LdT, n=46 | | ADV, n=2 | TDF, n=12 | | p value |
| n=196 | 29 (38.7)^a,b,c^ | | 44 (72.1)^a^ | | 29 (63)^b^ | | 0 (0) | 8 (66.7)^c^ | | 0.001 |
|  | HS (−), 40 | HS (+), 35 | HS (−), 25 | HS (+), 36 | HS (−), 23 | HS (+), 23 | − | HS (−), 5 | HS (+), 7 |  |
|  | 18 (45) | 11 (31.4) | 18 (72) | 26 (72.2) | 14 (60.9) | 15 (65.2) | − | 4 (80) | 4 (57.1) |  |
| p value | 0.334 | | 1.000 | | 1.000 | |  | 0.576 | |  |
| Age/gender- | LAM, n=46 | | ETV, n=32 | | LdT, n=27 | | ADV, n=2 | TDF, n=5 | | p value |
| matched, | 14 (30.4)^d,e^ | | 26 (81.3)^d^ | | 16 (59.3)^e^ | | 0 (0) | 2 (40) | | <0.001 |
| n=112 | HS (−), 24 | HS (+), 22 | HS (−), 16 | HS (+), 16 | HS (−), 12 | HS (+), 15 | − | HS (−), 3 | HS (+), 2 |  |
|  | 10 (41.7) | 4 (18.2) | 12 (75) | 14 (87.5) | 6 (50) | 10 (66.7) | − | 2 (66.7) | 0 (0) |  |
| p value | 0.159 | | 0.654 | | 0.452 | |  | 0.400 | |  |

Presented with number (%)

LAM, lamivudine; ADV, adefovir dipivoxil; LdT, Telbivudine; ETV, entecavir; TDF, tenofovir disoproxil fumarate; HS: hepatic steatosis

a, p<0.001; b, p=0.016; c, p=0.132; d, p<0.001; e, p=0.030

Supplementary Table 3. Comparison of baseline characteristics and treatment response among patients with different degrees of histological steatosis

| Steatosis* | Mild | Moderate | Severe | p |
| --- | --- | --- | --- | --- |
| No | 64 | 25 | 13 |  |
| Age at treatment, year | 43.7±10.6 | 41.4±12.0 | 40.5±9.5 | 0.608 |
| Male | 48 (75) | 23 (92) | 10 (77) | 0.198 |
| BMI, kg/m^2^ | 24.3±2.8 | 26.2±3.0 | 27.9±3.1 | 0.191 |
| Genotype  B  C | 29 (49.2)  30 (50.8) | 6 (25)  18 (75) | 5 (45.5)  6 (54.5) | 0.128 |
| Treatment-naïve | 50 (78.1) | 20 (80) | 12 (92.3) | 0.501 |
| Cirrhosis | 23 (35.9) | 12 (48) | 5 (38.5) | 0.577 |
| AST, U/L | 66 (47-87) | 49 (34-81) | 47 (36-71) | 0.070 |
| ALT, U/L | 125 (68-163) | 92 (56-142) | 81 (37-122) | 0.106 |
| Total bilirubin, mg/dL | 0.8 (0.6-1.0) | 0.8 (0.7-0.9) | 0.9 (0.7-1.1) | 0.607 |
| Platelet, 10^9^/L | 177 (152-217) | 192 (150-220) | 195 (162-229) | 0.620 |
| qHBsAg, log IU/mL | 3.7±0.9 | 3.7±0.6 | 3.5±0.5 | 0.757 |
| HBV DNA, log IU/mL | 7.6±1.2 | 7.1±1.3 | 6.6±1.0 | 0.433 |
| Treatment duration, m | 24.4 (12.3-48.6) | 24.3 (13.1-64.1) | 24.2 (12.1-36.3) | 0.565 |
| **Treatment response** | | | | |
| HBeAg seroclearance | 37 (57.8) | 14 (56) | 5 (38.5) | 0.438 |
| Virological response | 40 (62.5) | 17 (68) | 8 (61.5) | 0.875 |
| Age at e seroclearance | 47.3±10.8 | 42.6±11.8 | 40.8±8.8 | 0.419 |
| Time to seroclearance, m | 17.7 (9.8-41.6) | 15.2 (6-38.6) | 16 (11.2-32.9) | 0.898 |

Data were presented as mean ± S.D. or median (interquartile range) and number (%)

BMI, body mass index; AST, aspartate aminotransferase; ALT, alanine aminotransferase; qHBsAg, quantitative HBsAg

*Steatosis: mild, 5-33%; moderate, 33-66%; severe, >66%

Supplementary table 4. Baseline clinical characteristics among patients with lamivudine (LAM) and entecavir (ETV) treatment*

|  | LAM | ETV | LdT | p |
| --- | --- | --- | --- | --- |
| No | 75 | 61 | 46 |  |
| Age, year | 35 (28-42) | 44 (37-53) | 36 (29-43) | <0.001 |
| Male | 58 (77.3) | 43 (70.5) | 60 (65.2) | 0.337 |
| BMI, kg/m^2^ | 23.7 (22.0-27.0) | 23.9 (22.3-26.6) | 22.9 (20.1-25.4) | 0.107 |
| Genotype  B  C | 39 (52.7)  35 (47.3) | 23 (40.4)  34 (59.6) | 32 (71.1)  13 (28.9) | 0.008 |
| Treatment-naïve | 55 (73.3) | 51 (83.6) | 44 (95.7) | 0.007 |
| Cirrhosis | 17 (22.7) | 31 (50.8) | 1 (2.2) | <0.001 |
| AST, U/L | 61 (42-83) | 70 (40-112) | 60 (44-86) | 0.504 |
| ALT, U/L | 103 (77-155) | 106 (54-195) | 121 (68-176) | 0.871 |
| <2x ULN  2-5x ULN  >5x ULN | 14 (18.7)  48 (64)  13 (17.3) | 23 (37.8)  19 (31.1)  19 (31.1) | 12 (26.1)  23 (50)  11 (23.9) | 0.006 |
| Total bilirubin, mg/dL | 0.9 (0.8-1.2) | 0.7 (0.6-0.9) | 0.8 (0.6-0.9) | <0.001 |
| Platelet, 10^9^/L | 210 (164-237) | 181 (149-214) | 193 (172-218) | 0.064 |
| qHBsAg, log IU/mL | 3.9 (3.5-4.3) | 3.7 (3.3-4.1) | 4.1 (3.6-4.4) | 0.149 |
| HBV DNA, log IU/mL | 7.5 (6.8-8.0) | 7.4 (6.4-8.0) | 8.0 (7.6-8.6) | <0.001 |

Presented with median (interquartile range) or number (%)

*2 adefovir dipivoxil (ADV) and 12 tenofovir disoproxil fumarate (TDF) were not included
